# Medico-economic impact of enhanced rehabilitation after surgery: an exhaustive, nation-wide claims study

**DOI:** 10.1186/s12913-021-07379-z

**Published:** 2021-12-14

**Authors:** Frédéric Bizard, Thierry Boudemaghe, Laurent Delaunay, Lucas Léger, Karem Slim

**Affiliations:** 1 Financial Reporting & Audit Department, ESCP, Paris, France; 2 Department of Medical Informatics (S.I.M.M.E.R.), Nîmes University Hospital, Pl Pr Robert Debré, 30 029 Nîmes, France; 3 Desbrest Institute of Epidemiology and Public Health, Univ Montpellier, INSERM, Nîmes University Hospital, Montpellier, France; 4 MD. Department of Anaesthesia, General Clinic, Annecy, France; 5 MD. Department of Digestive Surgery, University Hospital Clermont-Ferrand, Clermont-Ferrand, France

**Keywords:** Enhanced rehabilitation after surgery, Care pathway, Cost-effectiveness, Quality of care

## Abstract

**Background:**

Study of the medico economic impact of enhanced rehabilitation after surgery (ERAS), by comparing the cost of patient care with or without ERAS, both from the point of view of the hospitals and the Social Security Health Insurance Program.

**Methods:**

Retrospective longitudinal study on matched data from March 1, 2019 to December 31, 2019. The data are extracted from the French prospective payment system. We studied 12 of the most commonly performed in ERAS business segments. The primary outcome was the reduction of the average length of hospital stay and its implications on production costs and excess capacity. We also studied the impact on hospital incomes and Social Security Insurance Program expenses. The potential gain in hospital days was computed by comparing the length of stay of ERAS and non-ERAS cases. The cost reduction was estimated using the mean number of avoidable days of hospitalization, and the mean cost of the stays obtained from the national cost study. Finally, we studied an approximation of the additional expense for the Social Security Health Insurance Program on costs standardized by applying public sector rates.

**Results:**

The average length of stay reduction attributed to ERAS is 1.45 (CI 95% 1.42 to 1.48) day per stay, translating to a cost reduction for the hospitals of € 1060 (CI 95% 995 to 1125) per patient and a total of €65 million (CI 95% 61 to 69). At the same time, the additional expenses for the Social Security Insurance Program can conservatively be approximated to € 1.6 million, breaking into a € 2.2 million increase partially compensated by cost savings of € 0.6 million over subsequent stays for complications. Overall, for each percent of additional ERAS activity over the scope of the study, the marginal cost reduction for the hospitals can be estimated to € 1.8 million (CI 95% 1.7 million to 2.0 million).

**Conclusions:**

Associated with previously known clinical benefits for the patients, these convincing results in terms of economic gain strongly support expanding the adoption of ERAS.

**Supplementary Information:**

The online version contains supplementary material available at 10.1186/s12913-021-07379-z.

## Background

Surgical procedures are a source of stress and responsible for hormonal, metabolic and physiological changes [1–3]. In this context, enhanced recovery programs (ERAS) aim to rapidly restore the patient’s pre-surgical physical and psychological capacities using a multidisciplinary approach to comprehensive patient care [4, 5]. The adjacent general purpose is to of course reduce morbidity and mortality, while improving postoperative recovery.

Medico-economic studies attribute a direct and substantial benefit to ERAS, with shorter length of stays without higher re-hospitalization rates [6–12].

As an example, a Canadian publication involving 6 hospitals [13] reported the deployment of ERAS in 1333 colorectal surgery patients. The decrease in the average length of stay (4.5 days versus 6.0 days, *p* < 0.001) and complications (RR 1.71 95% CI 2.5-2.1, *p* = 0.013) observed after the implementation of ERAS had an impact estimated by the authors between $ 2806 and $ 5898 per patient. A second study on colorectal surgery carried out at the Lausanne University Hospital [14] demonstrated a reduction in the average length of stay of three days and a net benefit per patient included in ERAS of around € 1700 per patient. The cost of implementing ERAS was offset by the reduction in pre- and postoperative costs.

In France, a feasibility study [15] was carried out at the Hospices Civils de Lyon (HCL- a public university hospital system) on five pilot sites for the deployment of improved recovery protocols after digestive (colorectal, pancreas, liver), orthopedic (hip, knee, shoulder cap) and urological (prostatectomy and bladder support, cystectomy) surgeries. The gains are essentially expressed as a shortening of two days for all the stays concerned, that is to say an estimated gain of 2300 to 3200 hospital days in 2012. The economic valuation of these gains amounts to € 202,000 for the first year of implementation and € 288,000 per year thereafter.

To date, the few ERAS medico-economic impact studies that exist are limited to a relatively small sample of patients, either by focusing on a few surgical specialties or on a few establishments, rendering generalization at higher levels difficult. Having a study on the whole of the activity would allow us to get an idea of the value of this care, both in terms of public health and healthcare funding.

In order to evaluate the global medico-economic impact of ERAS, we performed a nationwide study on the French prospective payment system claims database. This database provides a unique opportunity to study ERAS across a wide range of surgical specialties and a large population of around 67 million persons.

The main objective of the current study is therefore to assess the economic impact of ERAS in France by evaluating the decrease in the average length of stay, from which we will valorize the potential gain in hospital days, completed by an estimation of the additional cost for the French National Social Security Health Insurance program.

## Methods

### General outline of the study

This is a retrospective longitudinal study on matched data from March 1, 2019 to December 31, 2019. The data are extracted from the French prospective payment system [16]. They provide information describing every hospital stay occurring in France, in particular the DRG (known as the “GHM”: *Groupe Homogène de Malade*), diagnoses, procedures, ERAS, length of stay, gender and age of the patient.

The data are collected during the stay, especially pathologies and procedures are coded using terminologies. All hospital reimbursements are entirely based upon these data. Furthermore, these data are subject to quality and accuracy controls led by the French Social Security administration, potentially resulting in fines for those hospitals not respecting certain coding criteria, ensuring significant data quality.

The French law related to the research involving human participants (Law 2012–300 of March 5, 2012, modified by Order 2016–800 of June 16, 2016) does not apply in the context of these retrospective data. Neither the French Health and Safety Authority approval nor the French ethics committee approval is required.

Selection and segmentation of the activity were carried out according to defined inclusion criteria (detailed in following sections). The main economic impact of ERAS was assessed as the decrease in the average length of hospital stay (ALOS) and its implications on production costs and excess capacity. We also studied the impact on the income of hospitals and Social Security expenses.

### Definition of activity segments

This study was carried out on specific activity segments. The latter were designed by combining DRGs and procedures to define groups as homogenous as possible in terms of pathology and management strategies. To achieve this, we relied on procedure characteristics as defined for the grouping function and their hierarchical position in their coding terminology (*Classification Commune des Actes Médicaux*, CCAM[17]). We therefore combined characteristics simultaneously pertaining to procedure cost, complexity, techniques and medical strategy to create lists of similar procedures.

The resulting activity segments covered the main areas of interest defined in the study protocol [(i) Orthopedics: Total Knee Prosthesis, Total Hip Prosthesis, Herniated Disc (lumbar and cervical), Cruciate Ligament Arthroscopic Surgery; (ii) Digestive surgery: colorectal, bariatric; (iii) Gynecology: hysterectomy] as well as two other segments concerning the field of oncology [(iv) Major surgery for malignant tumor of the prostate; (v) Malignant tumor of the lung]. The corresponding hospital stays for each business segment were then extracted in order to examine their representativeness within their DRG, and the general fraction of the activity in ERAS represented.

### Definition of complications

For the follow-up of the patients, we selected some DRGs supposed to be a probable complication of a former stay for one of the business segments. This selection was done by a preliminary, global study of the intervals between stays depending on the DRGs they are grouped in. Couples of DRG are formed according to their statistical characteristics in terms of interval between hospitalizations. After this first step, the couples are assessed from a medical point of view, and the DRGs that are the most likely to be a complication of a former stay are selected as a potential complication. See Additional File 1 for detailed description of the constitution of the activity segments and the selection of complications.

### Eligibility criteria and matching

Adult hospital stays were retained (exhaustive sampling) as cases if they corresponded to one of the business segments with ERAS coded for a discharge date during the inclusion period. As ERAS cannot be coded for cases of in-hospital death, the latter were excluded. Indeed, a stay ending in death bearing the mention of ERAS is not eligible for reimbursement, and they are therefore systematically not coded as ERAS, thus inducing a bias.

In order to study comparable populations, ERAS and non-ERAS cases were matched according to activity segment, type of hospital, sex, age, and month of discharge (to avoid seasonality effects). Furthermore, to avoid a possible bias due to the selection of patients for ERAS or non-ERAS on the basis of their condition, matching also considered three additional disease severity measures: (i) the severity level of the DRG, (ii) the updated Charlson score [18], and (iii) the Charlson score comorbidity profile. Since the population was of ample size, we could perform the matching on a 1:1 basis on the exact values for each factor.

### Inclusion and follow-up period

The inclusion and follow-up periods were determined according to two factors: (i) a planned start date of March 1, 2019, and (ii) the onset of the Covid crisis, with the accompanying disruption of activity, in March 2020. The inclusion period thus corresponded to stays with an exit date between March 1 and December 31, 2019, with population-level follow-up lasting until February 2020, ensuring at least 2 months of follow-up for each stay. Individual follow-up lasted until re-hospitalization for surgical complications or re-hospitalization ending in death (with end of stay dates between March 1, 2019 and February 29, 2020). If none of the previous two events occurred, observations were right censored on February 29, 2020.

### Analysis

The difference in LOS between ERAS and non-ERAS cases was used to estimate the potential gain in hospital days, had all cases been carried out using ERAS. This calculation is made by bootstrap, for each business segment and for each hospital sector and globally. Secondly, using (a) the mean number of avoidable days of hospitalization, and (b) the mean cost of the stays obtained from the national cost study [19, 20], we estimated the cost reduction associated with each additional percentage of ERAS stays. Third, standardizing costs by applying public sector rates, we studied an approximation of the additional expense for the Social Security Health Insurance Program, with and without the presence of complications in the year following surgery. Since public rates are higher than private ones, this analysis aims at conservatively producing an information about a general trend if not an exact value. See Additional File 1 for precisions on the methods used to compute cost reduction for the hospitals and cost comparison for the National Health Insurance Program.

## Results

The overall cohort consists of 419,095 stays, subdivided into 78,119 (22.9%) ERAS stays, and 340,976 (67.1%) non-ERAS stays. Successful matching was achieved for 62,403 (79.9%) ERAS stays, matched with an equal number of non-ERAS stays. Two segments are discarded for the remaining analysis: a) colectomy without restoration of continuity presents too few ERAS stays and b) cruciate ligament arthroscopic surgery shows a too low ERAS rate.

For the remaining segments, the minimum matching rate is 26.0% (rectal resection, public sector), the maximum 91.5% (hysterectomy without malignant tumor, private sector). Apart from colectomy with restoration, the matching rates and generalizability are the highest for the most significant segments (total hip replacement, total knee replacement, herniated disk, hysterectomy without malignant tumor, obesity, malignant tumor of the prostate), which represent 292,371 (76.3%) of the retained population. The selected segments are listed in Table 1; see Additional File 1 for further precisions on the matching results.

**Table 1:** List of segments of activity studied

Segment	Sector 1 = Pub 2 = Pri	Selected stays				Matched ERAS stays	
		Total	Non ERAS	ERAS	% ERAS	Number of stays	% of matched ERAS stays
01-THR - Total Hip Replacement	/ 1	29,524	22,564	6960	30.8%	5592	80.3%
	/ 2	53,461	35,488	17,973	50.6%	15,107	84.1%
02-TKR -Total Knee Replacement	/ 1	31,478	24,800	6678	26.9%	5400	80.9%
	/ 2	57,224	39,992	17,232	43.1%	14,713	85.4%
03-TSR - Total Shoulder Replacement	/ 1	4112	3812	300	7.9%	193	64.3%
	/ 2	7634	6352	1282	20.2%	921	71.8%
04-HDS -Herniated Disc	/ 1	11,602	11,186	416	3.7%	337	81.0%
	/ 2	32,481	26,629	5852	22.0%	5054	86.4%
05-HCR -Cervical Herniation	/ 1	4769	4634	135	2.9%	86	63.7%
	/ 2	17,057	14,879	2178	14.6%	1822	83.7%
07-OBE - Obesity	/ 1	11,526	9993	1533	15.3%	1123	73.3%
	/ 2	17,487	13,251	4236	32.0%	3495	82.5%
08-COL-1 - Colectomy with Restoration of Continuity	/ 1	15,617	13,425	2192	16.3%	1094	50.0%
	/ 2	11,232	9873	1359	13.8%	769	56.6%
08-COL-2 -Colectomy w/out Restoration of Continuity	/ 1	2364	2243	121	5.4%	21	17.4%
	/ 2	746	720	26	3.6%	2	7.7%
09-RRC -Rectal Resection	/ 1	4873	3860	1013	26.2%	263	26.0%
	/ 2	5883	5094	789	15.5%	353	44.7%
10-HYS-1 - Hysterectomy w/out Malig Tum	/ 1	18,513	17,401	1112	6.4%	980	88.1%
	/ 2	14,177	12,761	1416	11.1%	1295	91.5%
10-HYS-2 - Hysterectomy for Malig Tum	/ 1	4158	3694	464	12.6%	266	57.3%
	/ 2	2222	1974	248	12.6%	128	51.6%
11-TPR - Malig Tum of the Prostate, major surgery	/ 1	6092	5435	657	12.1%	561	85.4%
	/ 2	8806	7414	1392	18.8%	1219	87.6%
12-TPM - Malig Tum of the Lung	/ 1	8495	7269	1226	16.9%	506	41.3%
	/ 2	4679	4406	273	6.2%	77	28.2%
14-ALC - Cruciate Ligament Arthroscopic Surgery	/ 1	5642	5558	84	1.5%	79	94.1%
	/ 2	27,241	26,269	972	3.7%	947	97.4%
**TOTAL**		**419,095**	**340,976**	**78,119**	**22.9%**	**62,403**	**79.9%**

### Avoidable days of hospitalization

ERAS stays were generally shorter than non-ERAS stays, with an average number of days saved ranging from 0.22 (cruciate ligament arthroscopic surgery) to 2.44 (malignant tumor of the lung). Overall, the mean length of stay reduction is of 1.45 days (CI 95% 1.42 to 1.48).

Conservatively projecting these values for each segment on the set of non-ERAS stays only eligible to matching, we can estimate that each 1% increase in ERAS usage applied to non-ERAS cases would result in a theoretical gain of 2590 (CI 95% 2540 to 2639) days of hospitalization. Table 2 shows the avoidable days of hospitalization for each segment.

**Table 2:** Avoidable days of hospitalization

Segment / Sector 1 = Pub 2 = Pri	N	Mean difference of length of stay	CI 95%	Days saved for additional 1% ERAS stays	CI 95%
01-THR / 1	5592	1.69	1.61 to 1.77	258.66	245.97 to 270.46
01-THR / 2	15,107	1.50	1.45 to 1.54	436.42	423.91 to 449.17
02-TKR / 1	5400	1.70	1.60 to 1.80	295.85	278.13 to 312.27
02-TKR / 2	14,713	1.57	1.52 to 1.61	514.49	498.50 to 530.44
03-TSR / 1	193	1.84	1.37 to 2.32	13.60	10.11 to 17.15
03-TSR / 2	921	1.44	1.28 to 1.61	47.82	42.41 to 53.32
04-HDS / 1	337	0.29	−0.04 to 0.63	5.22	−0.81 to 11.53
04-HDS / 2	5054	1.09	1.02 to 1.16	218.53	204.62 to 231.82
05-HCR / 1	86	1.53	0.86 to 2.24	3.71	2.08 to 5.43
05-HCR / 2	1822	1.26	1.10 to 1.41	109.93	96.24 to 123.50
07-OBE / 1	1123	0.91	0.79 to 1.03	43.37	37.40 to 48.78
07-OBE / 2	3495	1.10	1.04 to 1.16	105.39	99.71 to 111.57
08-COL-1 / 1	1094	2.02	1.57 to 2.49	43.25	33.68 to 53.46
08-COL-1 / 2	769	1.40	0.97 to 1.82	21.76	15.09 to 28.21
09-RRC / 1	263	1.74	0.56 to 2.81	5.74	1.85 to 9.32
09-RRC / 2	353	1.86	1.13 to 2.63	10.06	6.12 to 14.18
10-HYS-1 / 1	980	0.88	0.74 to 1.01	101.62	85.76 to 116.76
10-HYS-1 / 2	1295	0.79	0.67 to 0.91	74.51	63.31 to 86.38
10-HYS-2 / 1	266	0.33	−0.35 to 0.94	1.68	−1.79 to 4.79
10-HYS-2 / 2	128	1.36	0.77 to 1.95	3.06	1.72 to 4.40
11-TPR / 1	561	1.75	1.53 to 1.98	45.21	39.56 to 51.22
11-TPR / 2	1219	2.11	1.93 to 2.31	105.21	95.98 to 114.82
12-TPM / 1	506	1.58	1.14 to 2.03	14.42	10.44 to 18.59
12-TPM / 2	77	2.44	1.68 to 3.26	2.66	1.83 to 3.55
**TOTAL**	**61,354**	**1.45**	**1.42 to 1.48**	**2590.02**	**2539.90 to 2639.35**

### Per patient and hospital cost reduction

Valuation of the LOS saved per patient translates to an average gain of € 1060 (CI 95% 995 to 1125) per patient. These savings range from € 196 for hysterectomy for malignant tumor to € 1848 for major surgery for malignant tumor of the prostate. On average, this gain represents 31% of the price of a level 1 DRG (i.e. the minimum price for a DRG). It can amount to up to 56.7% of the price for a total shoulder replacement in the private sector, with a minimum of 5.5% of the price for hysterectomy for malignant tumor in the public sector. Over the matched stays, the total gain is € 65 million. As for the repartition between sectors, these gains correspond to 33.3% saving for the public sector and 66.7% savings for the private sector. These results involve a potential economic gain per additional percentage of ERAS of € 1.8 (CI 95% 1.7 to 2.0) million over the matched data. Table 3 shows the cost reduction for each segment.

**Table 3:** Cost reduction for hospital

Segment / Sector 1 = Pub 2 = Pri	Cost	Non- ERAS ALOS	Cost per day	Mean cost reduction per stay (€)	CI 95%	Cost reduction for additional 1% ERAS stays (€)	CI 95%
01-THR / 1	4780.12	6.88	694.95	1174.47	1116.85 to 1228.09	179,753.11	170,933.99 to 187,958.58
01-THR / 2	4848.39	6.80	712.89	1066.28	1035.71 to 1097.44	311,119.50	302,200.30 to 320,211.47
02-TKR / 1	5951.78	7.78	765.26	1303.05	1224.99 to 1375.36	226,404.27	212,841.23 to 238,968.70
02-TKR / 2	6083.25	7.70	789.67	1236.92	1198.48 to 1275.25	406,278.16	393,654.06 to 418,869.25
03-TSR / 1	4766.93	6.18	770.84	1418.91	1054.32 to 1789.32	10,485.71	7791.42 to 13,223.06
03-TSR / 2	4989.82	5.15	969.49	1397.99	1239.99 to 1558.97	46,357.24	41,118.09 to 51,695.41
04-HDS / 1	2635.25	4.21	625.95	179.28	27.86 to 395.68	3270.15	− 508.19 to 7217.19
04-HDS / 2	2221.21	3.72	596.72	651.78	610.30 to 691.42	130,401.97	122,102.41 to 138,332.02
05-HCR / 1	5214.23	7.27	717.36	1099.26	617.27 to 1609.90	2660.21	1493.78 to 3895.95
05-HCR / 2	4747.30	5.22	909.99	1146.14	1003.38 to 1287.60	100,035.26	87,575.17 to 112,381.96
07-OBE / 1	2895.90	4.00	723.59	660.54	569.58 to 743.01	31,382.35	27,060.80 to 35,300.19
07-OBE / 2	3406.49	4.24	803.02	880.13	832.66 to 931.70	84,632.83	80,068.63 to 89,592.24
08-COL-1 / 1	3923.23	12.92	303.59	611.86	476.48 to 756.24	13,130.57	10,225.27 to 16,228.81
08-COL-1 / 2	3692.20	11.26	328.01	459.62	318.63 to 595.90	7137.87	4948.30 to 9254.39
09-RRC / 1	5262.53	20.09	261.90	454.44	146.19 to 737.06	1504.21	483.88 to 2439.65
09-RRC / 2	4844.81	14.76	328.26	611.80	371.84 to 862.04	3303.69	2007.96 to 4655.03
10-HYS-1 / 1	2408.29	2.89	832.67	729.90	616.00 to 838.61	84,617.07	71,413.29 to 97,220.58
10-HYS-1 / 2	2195.00	3.29	667.18	525.70	446.67 to 609.49	49,710.26	42,237.57 to 57,633.34
10-HYS-2 / 1	3681.90	7.15	514.60	169.12	− 180.16 to 483.69	862.49	− 918.81 to 2466.84
10-HYS-2 / 2	3239.41	7.25	446.88	607.13	342.06 to 872.99	1366.04	769.63 to 1964.24
11-TPR / 1	5613.06	5.31	1056.46	1848.34	1617.55 to 2094.14	47,761.12	41,797.55 to 54,112.48
11-TPR / 2	4690.24	6.38	735.24	1552.93	1416.73 to 1694.84	77,351.40	70,567.36 to 84,420.06
12-TPM / 1	4414.12	10.94	403.49	636.63	460.88 to 820.54	5818.82	4212.49 to 7499.72
12-TPM / 2	4362.43	11.43	381.63	930.48	639.35 to 1244.00	1014.22	696.89 to 1355.96
**TOTAL / AVERAGE**				**1059.89**	**994.60 to 1124.74**	**1,826,358.53**	**1694,773.08 to** **1,956,897.12**

### Cost comparisons for stays from the points of view of the French social security health insurance program

There is a significantly different distribution of the costs in 8 out of 12 segments. Six segments are significantly more expensive for ERAS stays (total hip replacement, total knee replacement, total shoulder replacement, herniated disc, obesity, hysterectomy without malignant tumor), while two are significantly less expensive (rectal resection and malignant tumor of the lung). These eight segments generate an additional standardized cost of € 2.176 million. The additional standardized cost for the 12 segments amounts to € 2.161 million.

### The cost of complications

When taking into account the occurrence of re-hospitalization for complications in the year following surgery, two types of surgery were more expensive when associated with ERAS: (i) hysterectomy without malignant tumor (10-HYS-1), and (ii) malignant tumor of the prostate (11-TPR). One was less expensive (total knee replacement, 02-TKR).

However, when considering the number of complicated stays, the resulting effect on the balance between ERAS and non ERAS is inverted for malignant tumor of the prostate and total knee replacement: the latter ends up being positive (cost increase) while the former is negative (cost reduction). The cost reduction observed for malignant tumor of the prostate is so important (€ 625,000) that the total balance for these three segment is a cost reduction of € 467,000. Over the 12 segments, the balance for complicated stays is in favor of ERAS with a balance of € 592,000.

Finally, the global result over all 12 segments is an estimated increase of expenses of € 1.569 million with ERAS (€ 2.176 million partially compensated by € 592,000). These results are presented Table 4.

**Table 4:** Cost comparison between non-ERPs and ERPs for the Social Security, initial stays and initial and 1 year complication stays combined

	Initial Stay				Initial Stay + 1 Year Complication Stay			
Segment / ERAS (0: no – 1: yes)	N	Mean Cost	*p* value	Balance	N	Mean Cost	p value	Balance
01-THR / 0	20,699	4763.66	< 0.001	549,558.45	606	11,262.72	0.664	− 144,036.52
01-THR / 1	20,699	4790.21			590	11,324.02		
02-TKR / 0	20,113	5318.66	< 0.001	312,556.02	492	10,159.03	0.002	37,170.84
02-TKR / 1	20,113	5334.20			540	9324.84		
03-TSR / 0	1114	4562.71	< 0.001	133,078.44	23	11,248.17	0.794	83,878.01
03-TSR / 1	1114	4682.17			32	10,705.81		
04-HDS / 0	5391	3546.21	< 0.001	787,355.55	76	9934.07	0.55	35,821.84
04-HDS / 1	5391	3692.26			76	10,405.41		
05-HCR / 0	1908	6664.50	0,252	54,778.68	48	14,484.10	0.297	− 335,081.91
05-HCR / 1	1,908	6693.21			27	13,339.07		
07-OBE / 0	4618	4635.90	< 0.001	481,380.32	213	8307.75	0.236	159,371.85
07-OBE / 1	4618	4740.14			220	8767.83		
08-COL-1 / 0	1863	7493.21	0.488	−158,653.08	72	11,500.01	0.726	69,977.39
08-COL-1 / 1	1863	7408.05			73	12,301.07		
09-RRC / 0	616	10,035.91	0.023	− 154,905.52	10	12,576.70	0.202	180,379.00
09-RRC / 1	616	9784.44			20	15,307.30		
10-HYS-1 / 0	2275	3013.49	< 0.001	298,684.75	85	5291.91	0.004	120,738.51
10-HYS-1 / 1	2275	3144.78			94	6069.69		
10-HYS-2 / 0	394	7419.70	0.177	58,493.24	5	11,681.00	0.894	40,596.98
10-HYS-2 / 1	394	7568.16			9	11,000.22		
11-TPR / 0	1780	6050.11	0.377	30,687.20	269	7825.49	< 0.001	− 624,957.56
11-TPR / 1	1780	6067.35			175	8457.71		
12-TPM / 0	583	8520.58	< 0.001	−231,975.70	96	9835.43	0.698	− 215,656.16
12-TPM / 1	583	8122.68			76	9586.12		
		**Total**		**2,161,038.35**		**Total**		**− 591,797.73**

## Discussion

To our knowledge, this is the first nation-wide study of its kind, providing the largest-scope study on the medico-economic impact of ERAS to date.

### ERAS, an essential source of efficiency gains

The most characteristic economic impact of ERAS is the reduction in LOS, which is 1.45 days on average over the scope of the study, in line with the LOS reduction observed in the Canadian publication [13] and the French feasability study [15]. A 1% increase in ERAS results in 2590 days of hospitalization saved. Taking into account full costs, while conservatively excluding facility, operating room and critical care costs, the potential gain for such a 1% increase is € 1.8 million for the establishments involved (33% of which can be attributed to public establishments and 67% to private). Expanding ERAS by 10 and 50%, would thus translate to considerable gains estimated (for our matching exercise) at € 18 million and € 91 million, respectively. In addition, the latter does not include gains related to the excess bed capacity generated by ERAS.

Per-patient savings amount to a potential € 1060 on average, but with considerable variation between business segments. The latter represents an average of 31% of stay rates. The differences in business segments suggests a prioritization of the implementation of ERAS, with the largest potential benefits found in Orthopedics. Compared to previous French results [15], the average amount saved is notably higher. We posit that this can be explained by the fact that our study has been carried on 6 years later, in the context of a much wider adoption of ERAS, and progress in the efficiency and implementation of the protocols.

There are some additional costs for the Social Security Insurance Program in association with ERAS. However, when assessing such impacts using public-rate-standardized costs, the resulting € 1.6 million standardized impact is limited compared with the previously mentioned benefits. Overall, ERAS appears to generate substantial efficiency gains for institutions while providing major medical benefits and simultaneously reducing the production cost of stays and increasing bed capacities.

A key point of the ERAS economic model remains the general principle of adapting the medical means to the needs of each patient. By making the patient’s needs the backbone of the business model, ERAS optimize the allocation of resources while improving the quality of care. We are clearly in the process of productivity and efficiency gain.

### Key issues to address to move towards the generalization of ERAS

ERAS rate figures are low in France. Although we have selected the highest ERAS rate activities for this study, this rate varies from 1.5 to 30.8%, with a global 22.9% apart from the total hip replacement and the total knee replacement in the private sector, which culminates respectively at 50.6 and 43.1%. Due to the novelty of ERAS coding guideline, these values are probably underestimated but they reflect the road that remains to be covered to make ERAS the standard for surgery. ERAS is still a minority approach in surgery in France.

The Public Authorities have introduced a financial incentive for the establishments performing ERAS by taking into account comorbidities in the DRG determination even for short stays. The series of interviews conducted during this study shows that this incentive is not in the minds of all hospital directors, some of whom even fear a loss of income with the deployment of ERAS. Yet not only does ERAS improve revenues, it also significantly reduces production costs. There is therefore a need for improved communication to make this reality known to all establishments.

However, this will not be enough to get ERAS off the ground in France. Incentives must be put in place, in various ways depending on the choices of the establishments: investment in innovative medical equipment, recruiting of paramedical personnel, bonuses, etc. They must be materialized by a tangible improvement in working conditions and better professional recognition of the collective effort to successfully set up ERAS.

Although not all countries have made ERAS the standard for their surgical practice, France is lagging far behind in the development of this innovative technique. This delay is problematic because patients do not benefit from the optimal care that science allows today.

Under-deployment of ERAS is also a missed opportunity to significantly improve the financial accounts of institutions. In 2018, more than 50% of public hospitals and more than 30% of private clinics were in financial deficit [21]. The extension of ERAS in both sectors is therefore an important lever for bringing institutions back to a structurally profitable financial situation while improving the quality of patient care and the working environment of the teams.

Finally, the delay of ERAS deployment in France has an impact on the average length of stay in the country. A comparative analysis of OECD countries [22] shows that in 2018, the average length of stay in France is 8.8 days, compared to 7.4 days on average in the OECD, or 19% higher.

### Study limitations

As for all retrospective database studies, care must be taken to interpret the results with caution and to not assume patterns of causality.

A further limitation of this study resides in the fact that it is impossible to quantify any hospital-to-hospital variation in ERAS procedures that might exist, or the adherence thereof; a high level of commitment and proper observance is required to generate optimal ERAS impacts, and from this perspective, our results are likely conservative in nature. Secondly, the targeted ERAS codes were only implemented in March 2019. Though hospitals are incentivized to code ERAS properly, this might not always be the case (and again, our results are therefore likely conservative).

The third limitation involves the interpretation of economic results. The estimated economic gains are partly theoretical, in the sense that they pertain to an ideal situation. They represent a potential to be exploited in proportions depending on the establishments’ capacity to reorganize themselves to capture this gain.

Finally, these results would greatly benefit from a precise investigation of the costs in ulterior studies in both private and public sectors, in order to refine the savings estimation. Furthermore, A large scale, multicentric, field study remains to be carried out in order to assess the effective impact of ERAS on the efficiency of hospital care.

Nevertheless, the fact remains that this assessment of potential economic gain sets a maximum benchmark from which management can determine performance objectives. Juxtaposed with these limitations is the overwhelming strength of this study, i.e. its completeness within the French population. The large sample of activities, hospitals and patients of this study gives it a robustness superior to previous studies. The matching process, while decreasing the number of subjects, further ensures conservative and robust estimations.

## Conclusions

This study demonstrates a significant economic impact associated with ERAS deployment. Considering the significant drop in average length of stay, ERAS should become established as the standard for surgery in France and internationally.

## Supplementary Information

**Additional file 1.**

## Data Availability

The datasets generated and/or analysed during the current study are not publicly available due to terms of the French law since they are assumed to be re-identifiable (no aggregation strategy ensuring at least 10 individuals in each group) but are available from the corresponding author on reasonable request.

## Abbreviations

ALOS: Average Length of Stay
DRG: Diagnosis Related Group
ERAS: Enhanced Rehabilitation After Surgery
GHM: Group Homogène de Malade
