# Supplementary material for: Medico-economic impact of enhanced rehabilitation after surgery: an exhaustive, nation-wide claims study
Source: BMC Health Serv Res. 2021 Dec 14;21:1341. doi: 10.1186/s12913-021-07379-z (PMC8672636; doi:10.1186/s12913-021-07379-z)
Supplement: Supplementary file 1 — Additional file 1. [file 12913_2021_7379_MOESM1_ESM.docx]

**Medico-economic impact of enhanced rehabilitation after surgery: an exhaustive, nation-wide claims study**

**Supplemental material**

# List of segments of activity studied

We constructed relevant segments of activity by combining GHM (Groupe Homogène de Malades, equivalent to diagnostic related groups - DRG) and procedures. A GHM code is a 6-digit code. The 5 first digits are called the root, and the 6^th^ the severity level. As a general rule, the severity level ranges from 0 to 4, the price being higher for a higher level of severity.

It is thus possible to aggregate data according to GHM roots in order to study similar stays. For this study, we selected activity by combining:

- GHM roots;

- level of severity 0 to 2, (0 to 3 for colectomy without restoration of continuity, colectomy without restoration of continuity, rectal resection);

- selected procedures.

The use of specific procedures inside a given root ensures further homogeneity between ERAS and non-ERAS stays.

Two segments are discarded for further analysis due to insufficient ERAS stay count or percentage (colectomy without restoration of continuity and cruciate ligament arthroscopic surgery).

# Method of selection of potential complication GHM roots

The GHM roots retained as potential complication have been determined by a preliminary, general analysis of the statistical characteristics of inter-hospitalization delays.

Given a couple of GHM roots (A,B), we study

- M_i_ the median hospitalization delay between a stay grouped in A and a stay grouped in B;
- M_post_ The median post-A delay: time to new hospitalizations after a stay grouped in A (i.e. delay to the first, the second, … the n^th^ hospitalization after);
- M_pre_ the median pre-B delay: time to a stay grouped in A after a former hospitalization (i.e. delay to the last, the second to last, … the n^th^ hospitalization before).

Couples of GHM roots showing similar relations regarding their M_i_, M_post_ and M_pre_ are grouped together. After this first step, a medical interpretation of similar couples is conducted. The roots that are the most likely to be a complication of a former stay are then selected as a potential complication (table A1).

**Table A1 - List of potential complications for each business segment**

| **Code Acti** | **Lib Acti** |  |
| --- | --- | --- |
|  | **GHM5c** | **LibGHM5c2** |
| **01-THR** | **Total Hip Replacement** | |
|  | 08C22 | Interventions for revision of joint prostheses |
|  | 08C50 | Interventions on the hip and femur except recent trauma, age over 17 years |
|  | 08C61 | Osteo-articular infection, major surgery |
|  | 08K04 | Fracture of hip and femur: closed reduction |
|  | 08M05 | Fractures of the shaft, epiphysis or unspecified part of the femur |
|  | 08M08 | Sprains and dislocations of hip and pelvis |
|  | 08M36 | Musculoskeletal system: Symptoms and other medical referrals |
|  | 09C15 | Interventions on the skin, subcutaneous tissue or breasts for traumatic injuries |
|  | 09M03 | Trauma to the skin and subcutaneous tissue, age over 17 years |
|  | 09M07 | Other dermatological conditions |
|  | 16M11 | Other erythroïd disorders, age over 17 years |
|  | 18C02 | Surgery for infectious or parasitic diseases |
|  | 21C05 | Other interventions for injuries or procedural complications |
|  | 21M16 | Other iatrogenic complications not elsewhere classified |
|  |  |  |
| **02-TKR** | **Total Knee Replacement** | |
|  | 05M07 | Deep vein thrombophlebitis |
|  | 08C53 | Interventions on the knee for trauma |
|  | 08C61 | Osteo-articular infection, major surgery |
|  | 08M30 | Rheumatism and joint stiffness |
|  | 08M36 | Musculoskeletal system: Symptoms and other medical referrals |
|  | 09C15 | Interventions on the skin, subcutaneous tissue or breasts for traumatic injuries |
|  | 09M03 | Trauma to the skin and subcutaneous tissue, age over 17 years |
|  | 09M07 | Other dermatological conditions |
|  | 21C05 | Other interventions for injuries or procedural complications |
|  | 21C06 | Skin grafts or wound areas for lesions other than burns |
|  | 21M16 | Other iatrogenic complications not elsewhere classified |
|  |  |  |
| **03-TSR** | **Total Shoulder Replacement** | |
|  | 08C22 | Interventions for revision of joint prostheses |
|  | 08C35 | Interventions on the arm, elbow and shoulder |
|  | 08C61 | Osteo-articular infection, major surgery |
|  | 08C62 | Other procedures for osteo-articular infection |
|  | 08K02 | Musculoskeletal system disorders without operative procedure, with anesthesia, ambulatory care |
|  | 08M30 | Rheumatism and joint stiffness |
|  | 21C05 | Other interventions for injuries or procedural complications |
|  | 21M16 | Other iatrogenic complications not elsewhere classified |
|  |  |  |
| **04-HDS** | **Herniated Disc** | |
|  | 01C05 | Intervention on spine and rachis for neurological diseases |
|  | 01M09 | Pathologies affecting the spine and the rachis |
|  | 01M12 | Other nervous system pathologies |
|  | 08C61 | Osteo-articular infection, major surgery |
|  | 09M05 | Injury, infection and inflammation of the skin and subcutaneous tissues, age over 17 years |
|  | 09M07 | Other dermatological conditions |
|  | 18C02 | Surgery for infectious or parasitic diseases |
|  | 21C05 | Other interventions for injuries or procedural complications |
|  | 21M16 | Other iatrogenic complications not elsewhere classified |
|  |  |  |
| **05-HCR** | **Cervical Herniation** | |
|  | 01C05 | Intervention on spine and rachis for neurological diseases |
|  | 01M09 | Pathologies affecting the spine and the rachis |
|  | 01M12 | Other nervous system pathologies |
|  | 08C61 | Osteo-articular infection, major surgery |
|  | 08M38 | Sprains and dislocations of the rachis |
|  | 09M03 | Trauma to the skin and subcutaneous tissue, age over 17 years |
|  | 09M05 | Injury, infection and inflammation of the skin and subcutaneous tissues, age over 17 years |
|  | 09M07 | Other dermatological conditions |
|  | 21C05 | Other interventions for injuries or procedural complications |
|  | 21M16 | Other iatrogenic complications not elsewhere classified |
|  |  |  |
| **07-OBE** | **Obesity** |  |
|  | 04M10 | Pulmonary embolism |
|  | 04M13 | Pulmonary edema and respiratory failure |
|  | 04M17 | Pleural effusion |
|  | 05C18 | Other circulatory system procedures |
|  | 05M07 | Deep vein thrombophlebitis |
|  | 06C07 | Minor interventions on the small intestine and colon |
|  | 06C15 | Other interventions on the digestive tract apart from laparotomies |
|  | 06C20 | Interventions on the esophagus, stomach and duodenum for ulcers, age over 17 years |
|  | 06C21 | Other interventions on the digestive tract by laparotomy |
|  | 06M04 | Gastrointestinal hemorrhage |
|  | 06M09 | Other digestive system pathologies, age over 17 years |
|  | 06M12 | Abdominal pain |
|  | 06M18 | Digestive system: Symptoms and other medical referrals |
|  | 06M19 | Severe disorders of the digestive tract |
|  | 07C08 | Other interventions on the hepato-biliary and pancreatic system |
|  | 07M04 | Other liver diseases |
|  | 07M15 | Severe hepatic disorders except malignant tumors, cirrhosis and alcoholic hepatitis |
|  | 10M08 | Diabetic ketoacidosis and coma |
|  | 10M16 | Metabolic disorders, age over 17 years |
|  | 11M06 | Renal failure, without dialysis |
|  | 11M19 | Kidneys and urinary tract: Symptoms and other medical referrals |
|  | 16C03 | Other procedures for disorders of the blood and hematopoietic organs |
|  | 16M06 | Spleen disorders |
|  | 18C02 | Surgery for infectious or parasitic diseases |
|  | 18M04 | Fever of unknown etiology, age greater than 17 years |
|  | 18M07 | Septicemia, age over 17 years |
|  | 21C05 | Other interventions for injuries or procedural complications |
|  | 21M07 | Unspecified trauma, age over 17 years |
|  | 21M16 | Other iatrogenic complications not elsewhere classified |
|  |  |  |
| **08-COL-1** | **Colectomy with Restoration of Continuity** | |
|  | 06C20 | Interventions on the esophagus, stomach and duodenum for ulcers, age over 17 years |
|  | 06M04 | Gastrointestinal hemorrhage |
|  | 06M09 | Other digestive system pathologies, age over 17 years |
|  | 06M10 | Complicated peptic ulcer |
|  | 06M19 | Severe disorders of the digestive tract |
|  | 07M11 | Other non-malignant pancreatic conditions |
|  | 09C15 | Interventions on the skin, subcutaneous tissue or breasts for traumatic injuries |
|  | 09M05 | Injury, infection and inflammation of the skin and subcutaneous tissues, age over 17 years |
|  | 10M14 | Endocrine system: Symptoms and other medical referrals |
|  | 10M18 | Various nutritional disorders, age over 17 years |
|  | 12C12 | Major pelvic procedures in men for non-malignant conditions |
|  | 12M07 | Other infections and inflammations of the male reproductive system |
|  | 13C13 | Other interventions on the female reproductive system |
|  | 13M05 | Infections of the uterus and adnexa |
|  | 16C02 | Surgery of the spleen |
|  | 16M06 | Spleen disorders |
|  | 18M10 | Severe infectious diseases |
|  | 21C05 | Other interventions for injuries or procedural complications |
|  | 21M16 | Other iatrogenic complications not elsewhere classified |
|  |  |  |
| **09-RRC** | **Rectal Resection** | |
|  | 06M04 | Gastrointestinal hemorrhage |
|  | 06M08 | Other digestive system pathologies, age less than 18 years |
|  | 09K02 | Skin, subcutaneous tissue and breast disorders without operative procedure, with anesthesia, ambulatory care |
|  | 09M05 | Injury, infection and inflammation of the skin and subcutaneous tissues, age over 17 years |
|  | 13M05 | Infections of the uterus and adnexa |
|  | 18C02 | Surgery for infectious or parasitic diseases |
|  | 18M11 | Other infectious or parasitic diseases |
|  | 21C05 | Other interventions for injuries or procedural complications |
|  | 21M16 | Other iatrogenic complications not elsewhere classified |
|  |  |  |
| **10-HYS-1** | **Hysterectomy without Malignant Tumor** | |
|  | 06C15 | Other interventions on the digestive tract apart from laparotomies |
|  | 06C21 | Other interventions on the digestive tract by laparotomy |
|  | 06M06 | Bowel obstruction not due to a hernia |
|  | 06M09 | Other digestive system pathologies, age over 17 years |
|  | 06M12 | Abdominal pain |
|  | 06M19 | Severe disorders of the digestive tract |
|  | 09C15 | Interventions on the skin, subcutaneous tissue or breasts for traumatic injuries |
|  | 09M03 | Trauma to the skin and subcutaneous tissue, age over 17 years |
|  | 09M05 | Injury, infection and inflammation of the skin and subcutaneous tissues, age over 17 years |
|  | 09M05 | Injury, infection and inflammation of the skin and subcutaneous tissues, age over 17 years |
|  | 11M04 | Kidney and urinary tract infections, age over 17 years |
|  | 11M12 | Kidney and urinary tract signs and symptoms, age over 17 years |
|  | 11M16 | Other disorders of the kidneys and urinary tract, except those of diabetic origin, age over 17 years |
|  | 11M19 | Kidneys and urinary tract: Symptoms and other medical referrals |
|  | 13C13 | Other interventions on the female reproductive system |
|  | 13M04 | Other disorders of the female reproductive system |
|  | 13M05 | Infections of the uterus and adnexa |
|  | 13M06 | Other infections of the female reproductive system |
|  | 16C03 | Other procedures for disorders of the blood and hematopoietic organs |
|  | 16M11 | Other erythroïd disorders, age over 17 years |
|  | 18C02 | Surgery for infectious or parasitic diseases |
|  | 18M04 | Fever of unknown etiology, age greater than 17 years |
|  | 18M07 | Septicemia, age over 17 years |
|  | 21C05 | Other interventions for injuries or procedural complications |
|  | 21M16 | Other iatrogenic complications not elsewhere classified |
|  |  |  |
| **10-HYS-2** | **Hysterectomy for Malignant Tumor** | |
|  | 13M04 | Other disorders of the female reproductive system |
|  | 21C05 | Other interventions for injuries or procedural complications |
|  | 21K02 | Trauma, allergies and poisoning without surgery, with anesthesia, ambulatory care |
|  | 21M16 | Other iatrogenic complications not elsewhere classified |
|  | 23C02 | Surgical interventions with other reasons for using health services |
|  |  |  |
| **11-TPR** | **Malignant Tumor of the Prostate, major surgery** | |
|  | 06C15 | Other interventions on the digestive tract apart from laparotomies |
|  | 06C21 | Other interventions on the digestive tract by laparotomy |
|  | 06M06 | Bowel obstruction not due to a hernia |
|  | 06M12 | Abdominal pain |
|  | 06M18 | Digestive system: Symptoms and other medical referrals |
|  | 09M03 | Trauma to the skin and subcutaneous tissue, age over 17 years |
|  | 09M05 | Injury, infection and inflammation of the skin and subcutaneous tissues, age over 17 years |
|  | 11C08 | Other kidney and urinary tract interventions |
|  | 11M04 | Kidney and urinary tract infections, age over 17 years |
|  | 11M12 | Kidney and urinary tract signs and symptoms, age over 17 years |
|  | 11M16 | Other disorders of the kidneys and urinary tract, except those of diabetic origin, age over 17 years |
|  | 11M19 | Kidneys and urinary tract: Symptoms and other medical referrals |
|  | 12M05 | Other conditions of the male reproductive system |
|  | 12M06 | Acute prostatitis and orchitis |
|  | 16M09 | Other disorders of the reticuloendothelial or immune system |
|  | 18M04 | Fever of unknown etiology, age greater than 17 years |
|  | 18M07 | Septicemia, age over 17 years |
|  | 21C05 | Other interventions for injuries or procedural complications |
|  | 21M16 | Other iatrogenic complications not elsewhere classified |
|  |  |  |
| **12-TPM** | **Malignant Tumor of the Lung** | |
|  | 04M07 | Respiratory infections and inflammations, age over 17 years |
|  | 04M12 | Pneumothorax |
|  | 04M13 | Pulmonary edema and respiratory failure |
|  | 04M15 | Other respiratory system diagnoses |
|  | 04M16 | Thoracic trauma |
|  | 04M17 | Pleural effusion |
|  | 05K14 | Establishment of vascular accesses for cardiovascular diseases, stays of less than 2 days |
|  | 05M17 | Autres affections de l'appareil circulatoire |
|  | 09C02 | Skin grafts and / or wound trimming for skin ulcer or cellulitis |

# Matching result

Matching was performed over the following factors: activity segment, type of hospital, sex, age, and month of discharge, the severity level of the DRG, the updated Charlson score, and the Charlson score comorbidity profile (matching on each category composing the score). Since the population was of ample size, we could perform the matching on a 1:1 basis on the exact values for each factor.

The representativeness and generalizability of the matching process can be assessed by respectively studying the proportion of ERAS stays matched and the proportion of non ERAS matchable stays in table A2 below. Table A3 provides the mean LOS of ERAS / non ERAS matched stays.

**Table A2 – Matched and matchable stays for** **each business segment**

|  |  |  | **ERAS stays** | | | **Non ERAS stays** | | |
| --- | --- | --- | --- | --- | --- | --- | --- | --- |
| **Segment** | **Sector 1=Pub 2=Pri** | **Total** | **Matched ERAS stays** | **Total ERAS stays** | **% ERAS matched stays** | **Non ERAS matchable stays** | **Total non ERAS stays** | **% non ERAS matchable stays** |
| **01-THR - Total Hip Replacement** | /1 | 29,524 | 5,592 | 6960 | 80,3% | 15,305 | 22,564 | 67,8% |
|  | /2 | 53,461 | 15,107 | 17973 | 84,1% | 29,178 | 35,488 | 82,2% |
| **02-TKR - Total Knee Replacement** | /1 | 31,478 | 5,400 | 6678 | 80,9% | 17,375 | 24,800 | 70,1% |
|  | /2 | 57,224 | 14,713 | 17232 | 85,4% | 32,846 | 39,992 | 82,1% |
| **03-TSR - Total Shoulder Replacement** | /1 | 4,112 | 193 | 300 | 64,3% | 739 | 3,812 | 19,4% |
|  | /2 | 7,634 | 921 | 1282 | 71,8% | 3,316 | 6,352 | 52,2% |
| **04-HDS - Herniated Disc** | /1 | 11,602 | 337 | 416 | 81,0% | 1,824 | 11,186 | 16,3% |
|  | /2 | 32,481 | 5,054 | 5852 | 86,4% | 20,007 | 26,629 | 75,1% |
| **05-HCR - Cervical Herniation** | /1 | 4,769 | 86 | 135 | 63,7% | 242 | 4,634 | 5,2% |
|  | /2 | 17,057 | 1,822 | 2178 | 83,7% | 8,728 | 14,879 | 58,7% |
| **07-OBE - Obesity** | /1 | 11,526 | 1,123 | 1533 | 73,3% | 4,751 | 9,993 | 47,5% |
|  | /2 | 17,487 | 3,495 | 4236 | 82,5% | 9,616 | 13,251 | 72,6% |
| **08-COL-1 - Colectomy with Restoration of Continuity** | /1 | 15,617 | 1,094 | 2192 | 49,9% | 2,146 | 13,425 | 16,0% |
|  | /2 | 11,232 | 769 | 1359 | 56,6% | 1,553 | 9,873 | 15,7% |
| **08-COL-2 -Colectomy w/out Restoration of Continuity** | /1 | 2,364 | 21 | 121 | 17,4% | 29 | 2,243 | 1,3% |
|  | /2 | 746 | 2 | 26 | 7,7% | 2 | 720 | 0,3% |
| **09-RRC - Rectal Resection** | /1 | 4,873 | 263 | 1013 | 26,0% | 331 | 3,860 | 8,6% |
|  | /2 | 5,883 | 353 | 789 | 44,7% | 540 | 5,094 | 10,6% |
| **10-HYS-1 - Hysterectomy without Malignant Tumor** | /1 | 18,513 | 980 | 1112 | 88,1% | 11,593 | 17,401 | 66,6% |
|  | /2 | 14,177 | 1,295 | 1416 | 91,5% | 9,456 | 12,761 | 74,1% |
| **10-HYS-2 - Hysterectomy for Malignant Tumor** | /1 | 4,158 | 266 | 464 | 57,3% | 510 | 3,694 | 13,8% |
|  | /2 | 2,222 | 128 | 248 | 51,6% | 225 | 1,974 | 11,4% |
| **11-TPR - Malignant Tumor of the Prostate, major surgery** | /1 | 6,092 | 561 | 657 | 85,4% | 2,584 | 5,435 | 47,5% |
|  | /2 | 8,806 | 1,219 | 1392 | 87,6% | 4,981 | 7,414 | 67,2% |
| **12-TPM - Malignant Tumor of the Lung** | /1 | 8,495 | 506 | 1226 | 41,3% | 914 | 7,269 | 12,6% |
|  | /2 | 4,679 | 77 | 273 | 28,2% | 109 | 4,406 | 2,5% |
| **14-ALC - Cruciate Ligament Arthroscopic Surgery** | /1 | 5,642 | 79 | 84 | 94,0% | 448 | 5,558 | 8,1% |
|  | /2 | 27,241 | 947 | 972 | 97,4% | 14,367 | 26,269 | 54,7% |

**Table A3 – Mean and median LOS of matched stays for** **each business segment**

|  |  | **ERAS stays LOS** | | **Non ERAS stays LOS** | |
| --- | --- | --- | --- | --- | --- |
| **Segment** | **Sector 1=Pub 2=Pri** | **Mean** | **Median** | **Mean** | **Median** |
| **01-THR - Total Hip Replacement** | /1 | 3,2 | 3 | 4,9 | 5 |
|  | /2 | 2,9 | 3 | 4,4 | 4 |
| **02-TKR - Total Knee Replacement** | /1 | 4,2 | 4 | 5,9 | 6 |
|  | /2 | 3,7 | 3 | 5,2 | 5 |
| **03-TSR - Total Shoulder Replacement** | /1 | 2,7 | 3 | 4,6 | 4 |
|  | /2 | 2,4 | 2 | 3,9 | 3 |
| **04-HDS - Herniated Disc** | /1 | 3,2 | 3 | 3,5 | 3 |
|  | /2 | 1,8 | 1 | 2,9 | 3 |
| **05-HCR - Cervical Herniation** | /1 | 3,9 | 4 | 5,5 | 5 |
|  | /2 | 3,0 | 3 | 4,3 | 4 |
| **07-OBE - Obesity** | /1 | 2,4 | 2 | 3,3 | 3 |
|  | /2 | 2,0 | 2 | 3,1 | 3 |
| **08-COL-1 - Colectomy with Restoration of Continuity** | /1 | 6,5 | 5 | 8,6 | 7 |
|  | /2 | 6,1 | 5 | 7,5 | 6 |
| **08-COL-2 -Colectomy w/out Restoration of Continuity** | /1 | 10,6 | 10 | 13,7 | 13 |
|  | /2 | 5,5 | 5,5 | 10,0 | 10 |
| **09-RRC - Rectal Resection** | /1 | 9,1 | 7 | 10,8 | 9 |
|  | /2 | 7,0 | 6 | 8,9 | 8 |
| **10-HYS-1 - Hysterectomy without Malignant Tumor** | /1 | 1,9 | 2 | 2,8 | 3 |
|  | /2 | 2,2 | 2 | 3,0 | 3 |
| **10-HYS-2 - Hysterectomy for Malignant Tumor** | /1 | 4,7 | 3 | 5,0 | 4 |
|  | /2 | 3,3 | 2,5 | 4,7 | 4 |
| **11-TPR - Malignant Tumor of the Prostate, major surgery** | /1 | 2,7 | 2 | 4,5 | 4 |
|  | /2 | 3,1 | 3 | 5,2 | 5 |
| **12-TPM - Malignant Tumor of the Lung** | /1 | 5,3 | 5 | 6,9 | 6 |
|  | /2 | 4,1 | 3 | 6,5 | 6 |
| **14-ALC - Cruciate Ligament Arthroscopic Surgery** | /1 | 0,9 | 1 | 1,8 | 1 |
|  | /2 | 0,5 | 0 | 0,7 | 0 |

# Computation of avoidable days of hospitalization

In order to obtain an estimate of the average difference of length of stay between non ERAS and ERAS stays, we perform a boostrap analysis on the matched stays, globally and by segment.

The booststrap analysis is carried on 1,000 samples extracted from the original population. As required by this technique, the sample size is equal to the population size, and the sampling is realized with replacement.

We thus obtain an estimate of the mean difference of length of stay, or average number of potentially avoidable days of hospitalization, along with a 95% confidence interval.

# Computation of cost reduction

Using a) the average number of potentially avoidable days of hospitalization per stay (i.e. the mean difference of length of stay, see table A3), b) the mean cost of the stays obtained from the national costs study (Etude National Commune des Coûts) [1, 2], we can estimate a cost reduction for each additional percentage of ERAS stays.

The data of the national costs study detail costs for each GHM and GHM root, and by expenditure item (e.g. operating room expenses, facility expenses). In order to proceed to a sound, conservative estimation, we compute an adjusted cost by deducing the following expenditure item from the total cost:

- facility related costs (i.e. maintenance)

- operating room

- critical care

Thus eliminating expenses directly linked to the surgical procedure and post surgical cares.

Considering the non-ERAS stays average length of stay, we can compute a mean cost per day (MCD). By multiplying MCD by the average number of potentially avoidable days of hospitalization, we obtain the cost reduction per stay. Again, in order to be conservative, we use the level 1 DRG adjusted cost for each segment for this estimation.

It is thus possible to estimate the cost reduction per additional percentage of ERAS stays (table A4). This calculation is made for each segment and by sector.

# Cost comparison for the Social Security

This analysis is performed by comparing the total cost for the Social Security of ERAS and non-ERAS stays. The total cost includes any supplements for critical care, daily packages for long stays, etc. Since public and sector rates are different, we apply public sector rates to all stays in order to standardize the costs.

The comparison is carried out a) for the initial stays then b) for the initial stays combined with their ulterior stay for complication, if any, occurring during the first year after the initial stay (table A5).

**Comparison a)** shows a significantly different distribution of the cost of ERAS and non-ERAS stays in 8 out of the 12 segments retained for analysis.

For six of them, ERAS is significantly more expensive for ERAS stays:

- 01-THR;

- 02-TKR;

- 03-TSR;

- 04-HDS;

- 07-OBE;

- 10-HYS-1.

Two of them are significantly less expensive for ERAS stays:

- 09-RRC;

- 12-TPM.

These eight segments generate an additional standardized cost of € 2.175 million. The additional standardized cost for the 12 retained segments amounts to € 2.160 million.

**Comparison b)** shows a significantly different distribution of the cost of the combined initial and ulterior stay for three segments. Two are more expensive (10-HYS-1, 11-TPR) and one is less expensive (02-TKR).

These three segments generate a standardized cost reduction of € 0.5 million. For the 12 retained segments, the result is still in favor of ERAS with a € 0.6 million balance.

**Overall balance**

For the 12 segments, the overall balance is an additional standardized cost of € 1.6 million, conservatively estimated by € 2.2 million - € 0.6 million.

**References**

1. ENC MCO | Stats ATIH. https://www.scansante.fr/applications/enc-mco. Accessed 7 Mar 2021.

2. ENC MCO Données 2019 | Publication ATIH. https://www.atih.sante.fr/enc-mco-donnees-2019. Accessed 7 Mar 2021.
